# Delayed Venous Thromboembolism Diagnosis and Mortality Risk

**DOI:** 10.1001/jamanetworkopen.2025.33928

**Published:** 2025-09-26

**Authors:** Min-Jeoung Kang, Richard Schreiber, Veysel Karani Baris, Frank Chang, Shadi Hijjawi, Alice Kim, John Novoa-Laurentiev, Tim Nye, Stuart Lipsitz, Khalid Nawab, Michael Sainlaire, Wenyu Song, Ania Syrowatka, Gregory Piazza, Tien Thai, Li Zhou, Lipika Samal, David W. Bates, Patricia C. Dykes

**Affiliations:** 1 Department of Medicine, Division of General Internal Medicine, Brigham and Women’s Hospital, Boston, Massachusetts; 2 Harvard Medical School, Boston, Massachusetts; 3 Penn State Health, Hershey, Pennsylvania; 4 Faculty of Nursing, Dokuz Eylul University, Izmir, Turkey; 5 MassGeneral Brigham, Boston, Massachusetts; 6 Oracle Health, Kansas, Missouri; 7 Division of Cardiovascular Medicine, Brigham and Women’s Hospital, Boston, Massachusetts; 8 Department of Health Policy and Management, Harvard T. H. Chan School of Public Health, Boston, Massachusetts

## Abstract

**Importance:**

Nonspecific symptoms of venous thromboembolism (VTE) hinder timely recognition, and no automated tools exist to monitor diagnostic delay.

**Objective:**

To evaluate rates of diagnostic delay of VTE (DOVE) at 2 health care systems using different electronic health record (EHR) systems, to categorize missed diagnoses, and to assess the association of diagnostic delays with 30-day all-cause mortality.

**Design, Setting, and Participants:**

This diagnostic study used retrospective EHR data from Mass General Brigham (MGB) and Penn State Health (PSH). For MGB, data from 2016 to 2021 were extracted, whereas for PSH, data from 2019 to 2022 data were used. An electronic clinical quality measure (eCQM) was developed and used to quantify DOVE. The eCQM analyses were conducted from 2020 to 2022 at MGB and from 2022 to 2023 at PSH; mortality analyses were conducted from 2023 to 2024. Patients with VTE and documented VTE-related symptoms in primary care were included.

**Exposure:**

Diagnosis of VTE.

**Main Outcomes and Measures:**

The primary outcomes were DOVE rates and 30-day all-cause mortality by time to diagnosis. DOVE rates were calculated using thresholds of greater than 24 hours and greater than 72 hours, validated through EHR review. Missed opportunities were categorized into system, practitioner, patient, and other unclassified factors. Thirty-day all-cause mortality and risk ratios (RRs) were compared between timely (≤24 hours) and delayed (>24 hours) diagnosis.

**Results:**

A total of 3525 patients were included; 3281 patients (mean [SD] age, 65.95 [15.14] years; 1692 [51.57%] female) were from MGB, and 244 patients (mean [SD] age, 65.34 [16.76] years; 128 [52.46%] female) were from PSH. DOVE eCQM rates using the greater than 24-hour definition were 79.43% (95% CI, 78.00%-81.00%) for MGB and 82.38% (95% CI, 77.00%-87.00%) for PSH. The DOVE rate using the greater than 72-hour definition was 69.89% (95% CI, 68.00%-71.00%) for MGB and 71.31% (95% CI, 65.00%-77.00%) for PSH. Practitioner-related delays were most common. At MGB, mortality increased from 17 deaths (2.52%) for timely diagnosis to 217 deaths (8.33%) for delayed diagnoses, with an RR of 3.31 (95% CI, 2.03-5.38). At PSH, mortality increased from 2 deaths (4.65%) to 12 deaths (5.97%) with an RR of 1.28 (95% CI, 0.30-5.53). Diagnosis at less than 24 hours was often associated with missed pulmonary embolism leading to death.

**Conclusions and Relevance:**

In this diagnostic study of delayed VTE diagnosis, consistently high DOVE rates and associated increases in all-cause mortality support the use of the DOVE eCQM tool to guide quality improvement efforts at institutional, regional, and national levels.

## Key Points

**Question:**

What is the rate of diagnostic delay of venous thromboembolism (>24 and >72 hours) across 2 health care systems according to an electronic clinical quality measure, and is there an association between diagnostic delay and 30-day all-cause mortality?

**Findings:**

In this diagnostic study including 3525 patients, diagnostic delays exceeded 24 hours in 79.43% of cases at the first institution and 82.38% of cases at the second institution; delays exceeded 72 hours in 69.89% of cases at the first institution and 71.31% of cases at the second institution. Delayed VTE diagnoses were associated with higher 30-day all-cause mortality.

**Meaning:**

These findings suggest that the electronic clinical quality measure was able to quantify delays in diagnosing VTE across care settings and could be used to guide quality improvement efforts.

## Teaser

This diagnostic study examines rates of diagnostic delay of venous thromboembolism at 2 health care systems and assesses the association of such diagnostic delays with 30-day all-cause mortality.

## Introduction

Venous thromboembolism (VTE) is a common, preventable public health problem affecting approximately 300 000 to 600 000 individuals in the US each year.^1^ VTE includes pulmonary embolism (PE) and/or deep vein thrombosis (DVT). The 30-day mortality rate has been reported to be up to 23%,^2,3,4^ so early detection and timely treatment are essential for preventing complications, including mortality. However, nonspecific VTE clinical signs and symptoms make timely recognition difficult, leading to frequently missed or delayed VTE diagnosis in patients who present to primary care with symptoms.^5,6,7,8^ There is a lack of automated measures to systematically quantify and routinely monitor this problem in outpatient settings, and no quality metric has defined the optimal time frame from the presentation of VTE symptoms in primary care or other outpatient settings to diagnosis.

Our team developed an electronic clinical quality measure (eCQM) that uses electronic health record (EHR) data to measure diagnostic delay of VTE (DOVE) in primary care settings,^9^ defined as a diagnosis of VTE occurring between 24 hours and 30 days following a primary care visit where VTE symptoms were present. The DOVE eCQM was endorsed by the 2023 Partnership for Quality Measurement Patient Safety Standing committee.^10^ However, it was not approved for the Centers for Medicare & Medicaid Services (CMS) Measures Under Consideration list because of feasibility concerns specifically related to the greater than 24-hour definition of delayed VTE diagnosis, citing challenges in accounting for weekends, holidays, and the additional time required to complete diagnostic testing.^11^

This study aims to analyze delayed VTE diagnosis using both greater than 24-hour and greater than 72-hour definitions, categorize missed diagnostic opportunities, and examine the potential association of VTE diagnostic delay in primary care with all-cause mortality. We specifically aimed to (1) assess the feasibility of implementing the DOVE eCQM in geographically distant integrated care systems using different EHR systems, (2) compare DOVE eCQM rates in the 2 systems using both greater than 24-hour and greater than 72-hour definitions, (3) validate the DOVE eCQM with experts to categorize the cause of missed diagnostic opportunities, and (4) investigate the association between delays in VTE diagnosis and 30-day all-cause mortality.

## Methods

### Study Settings

In this diagnostic study, we conducted testing within 2 integrated delivery networks: Mass General Brigham (MGB; Boston, Massachusetts) and Penn State Health (PSH; Hershey, Pennsylvania). MGB uses the Epic system, whereas PSH uses the Oracle/Cerner EHR system. Primary care practices using the enterprise EHR for up to 5 years were included in the study. For MGB, data from 2016 to 2021 were extracted, whereas for PSH, data from 2019 to 2022 data were used. The eCQM analyses were conducted from 2020 to 2022 at MGB and from 2022 to 2023 at PSH; mortality analyses were conducted from 2023 to 2024. Race and ethnicity were patient-reported and documented in the EHR using predefined categories (ie, African American or Black, Hispanic, White, and other, which includes American Indian or Alaska Native, Asian, and race self-reported as other) to assess potential disparities in outcomes. The study and waiver of informed consent for data use were approved by the institutional review boards of MGB and PSH; consent was waived because data were deidentified, in accordance with 45 CFR §46. This study follows the Transparent Reporting of a Multivariable Prediction Model for Individual Prognosis or Diagnosis (TRIPOD) reporting guideline by transparently describing the development, validation, and evaluation of the DOVE eCQM using multivariable EHR data to identify delayed VTE diagnosis and associated outcomes.

### The DOVE eCQM Specifications

The DOVE eCQM consists of 2 components: first, an EHR-derived phenotyping algorithm was used to identify incident cases of VTE (Figure and eAppendix 1 in Supplement 1), as described elsewhere.^12^ This algorithm required a combination of *International Statistical Classification of Diseases and Related Health Problems, Tenth Revision* billing codes, *Current Procedural Terminology* scan codes, and RxNorm anticoagulant treatment codes to identify patients with a VTE diagnosis; second, a natural language processing (NLP) algorithm^13^ (eAppendix 2 in Supplement 1) was used to identify 29 VTE signs and symptoms in primary care visit notes (eg, negative predictive value [NPV] or specificity). The DOVE eCQM denominator includes all adult patients aged 18 years and older who received a diagnosis of VTE within 30 days of the index visit during which VTE-related signs and/or symptoms were reported. Symptoms recorded during the primary care visit mark the index for tracking time to diagnosis. For patients with multiple encounters, the index visit is defined as the first encounter in which VTE-related signs and symptoms are documented in the clinical note. Excluded were patients with an eligible VTE event within 6 months of the qualifying VTE event, and those receiving hospice or palliative care within 90 days of the index visit. The numerator includes those who received a diagnosis greater than 24 hours after the visit. The DOVE eCQM is expressed as a rate with lower rates indicative of better performance (see eAppendix 1 in Supplement 1 for a detailed explanation of the construction of the numerator and denominator).

**Figure.**
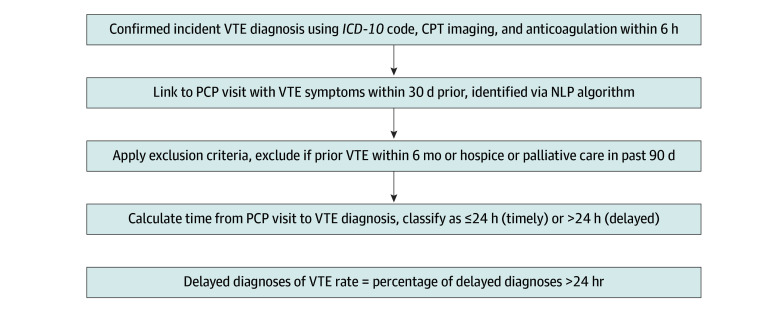
Process for Identifying Eligible Patients and Incident Cases of Venous Thromboembolism (VTE) Figure illustrates the steps for identifying eligible patients for the diagnostic delay of VTE (DOVE) electronic clinical quality measure (eCQM). Incident VTE cases were defined using a validated algorithm requiring diagnostic, imaging, and treatment criteria. Each case was linked to a primary care practitioner (PCP) visit within the prior 30 days containing VTE-related symptoms, identified via natural language processing (NLP). Patients aged 18 years or older with no prior VTE and not receiving end-of-life care were included. The DOVE rate reflects the proportion diagnosed more than 24 hours after the index visit. *CPT* indicates *Current Procedural Terminology*; *ICD-10*, *International Statistical Classification of Diseases and Related Health Problems, Tenth Revision.*

### The DOVE eCQM Performance Calculation

We first evaluated the reliability of data by calculating the frequency of the required data elements needed for the measure. Subsequently, we calculated DOVE rates and 95% CIs, including for the greater than 24-hour time frame and for a greater than 72-hour time frame as requested by CMS. Using national quality measure submission standards,^14,15^ we calculated measure performance scores using minimum, median, maximum, and quartiles (quartiles 1 and 3), focusing on primary care practices that have more than 20 patients with a VTE diagnosis during the study period.

### Validation of DOVE eCQM

Trained clinicians, 2 from each site (MGB, M.J.K. and V.K.B.; PSH, R.S. and S.H.), completed measure validity testing through an EHR review. Manual EHR review classifications (ie, criterion standard) were compared with those produced by the eCQM, and the positive predictive value (PPV), NPV, and accuracy were calculated. In addition, reviewers classified missed opportunities for timely diagnosis according to practitioner, medical system, and patient factors or unclassified (unable to classify).^16,17^ A total of 193 cases were reviewed from both sites, including 95 delayed cases and 98 nondelayed cases. The required sample size was 146 cases.^18^ To ensure interrater reliability, 2 clinician reviewers from each site (MGB, M.J.K. and V.K.B.; PSH, R.S. and S.H.) independently reviewed a 10% random sample of the total reviewed cases, discussed their findings to reach consensus, and then proceeded to review the remaining cases.

### Statistical Analysis

Thirty-day all-cause mortality was defined as death from any cause occurring within 30 days of the confirmed VTE diagnosis. We calculated 30-day all-cause mortality and risk ratios (RRs) associated with time to VTE diagnosis for both sites. RRs were calculated relative to timely diagnosis compared with those delayed diagnosis cases. Mortality data were identified using death information documented in the EHR, and time to death was calculated from the time of the VTE confirmed diagnosis to death. The RRs for death within 30 days were estimated using generalized estimating equations for binary outcomes (death within 30 days) clustering by practice, with a log-link and time to VTE as the only covariate. The log-link allows us to exponentiate the regression coefficient to obtain the RR, as well as obtain 95% CIs and 2-sided *P* values (*P* < .05 was statistically significant). The analyses were performed using R statistical software version 4.4.1 (R Project for Statistical Computing) and Excel software version 2506 (Microsoft Corporation).

To further understand the association between VTE and VTE-related mortality, we subdivided our analysis by VTE type as either PE or non-PE (DVT and others) using *International Statistical Classification of Diseases and Related Health Problems, Tenth Revision* codes. In addition, we conducted supplementary EHR reviews of deceased patients from a random sample of 40 cases at MGB and 14 cases at PSH, including both those with timely and delayed VTE diagnoses. These reviews focused on identifying the type of VTE whether it was PE or DVT, and whether the death was related to the VTE.

## Results

The study included 3525 adults with VTE and documented symptoms in primary care; 3281 patients (mean [SD] age, 65.95 [15.14] years; 1692 [51.57%] female) were from MGB, and 244 patients (mean [SD] age, 65.34 [16.76] years; 128 [52.46%] female) were from PSH. With regard to race and ethnicity, 305 patients (8.65%) were African American or Black, 223 (6.38%) were Hispanic, 2896 (82.16%) were White, and 324 (9.19%) were other races. Most had public (1966 patients [55.77%]) or private (1543 patients [43.77%]) insurance, and 3272 (92.82%) reported English as their first language (Table 1).

**Table 1.** Descriptive Statistics of Denominator Demographics

Characteristic	Patients, No. (%)	
	MGB (n = 3281)^a^	PSH (n = 244)^a^
No. of primary care practices	187	19
Time to diagnosis, d		
Mean (SD)	11.90 (9.80)	11.50 (9.48)
Median (IQR)	10.87 (1.79-20.97)	9.97 (2.24-20.19)
Age		
Age at VTE diagnosis, mean (SD), y	65.95 (15.14)	65.34 (16.76)
Age >65 y	1910 (58.21)	139 (56.97)
Sex		
Female	1692 (51.57)	128 (52.46)
Male	1589 (48.43)	116 (47.54)
Self-reported race		
African American or Black	285 (8.69)	20 (8.20)
White	2692 (82.05)	204 (83.61)
Other^b^	304 (9.27)	20 (8.2)
Self-reported ethnicity		
Hispanic	216 (6.58)	7 (2.87)
Non-Hispanic	3003 (91.53)	234 (95.90)
Multiple	0	3 (1.23)
Missing or declined	62 (1.89)	0
Insurance type		
Public insurance	1793 (54.65)	173 (70.90)
Private insurance	1476 (44.99)	67 (27.46)
Other insurance^c^	12 (0.37)	4 (1.64)
English as a first language	3034 (92.47)	238 (97.54)
Annual household income (via zip code), mean (SD), $	74 183 (27 330)	58 619 (10 230)
VTE signs and symptoms, mean (SD), No.	2.26 (1.32)	2.8 (1.81)

Abbreviations: MGB, Mass General Brigham; PSH, Penn State Health; VTE, venous thromboembolism.

^a^ The number of encounters with a VTE diagnosis and documented signs and symptoms in primary care visit notes constitutes the diagnostic delay of VTE electronic clinical quality measure–eligible denominator.

^b^ Other racial category includes American Indian or Alaska Native, Asian, and race self-reported as other.

^c^ Other insurance category includes self-pay and free care.

### Data Elements Availability and Frequency Calculation

Data needed to calculate the measures were available in the EHR databases of both sites. A total of 1.5% of ethnicity data were missing among MGB encounters, whereas no data elements were missing for PSH encounters. We identified 3281 DOVE eCQM–eligible denominator encounters at MGB and 244 at PSH, defined as encounters with both a VTE diagnosis and documented VTE-related symptoms in primary care visit notes. These encounters took place across 187 primary practices at MGB and 19 practices at PSH (Table 1).

### The DOVE eCQM Performance Calculation

The rate of VTE diagnostic delay of adults in primary care using the greater than 24-hour definition was 79.43% (95% CI, 78.00%-81.00%) for MGB and 82.38% (95% CI, 77.00%-87.00%) for PSH. The DOVE rate using the greater than 72-hour definition was 69.89% (95% CI, 68.00%-71.00%) for MGB and 71.31% (95% CI, 65.00%-77.00%) for PSH. Primary care practices group-level DOVE performance scores ranged from 0.59% to 0.95% for the 24-hour time frame and from 0.43% to 0.90% for the 72-hour time frame at MGB. At PSH, the DOVE performance scores ranged from 0.72% to 0.95% for the 24-hour time frame and 0.64% to 0.85% for the 72-hour time frame (Table 2). There were variable but consistently high DOVE rates across the different integrated care delivery system locations (Table 2).

**Table 2.** Diagnostic Delay of VTE Electronic Clinical Quality Measure Performance Scores by VTE Diagnosis Time Frames

Variable	Score, %^a^			
	MGB (n = 33)		PSH (n = 6)	
	24 h	72 h	24 h	72 h
Minimum	0.59	0.43	0.72	0.64
Quartile 1	0.70	0.60	0.79	0.68
Median	0.80	0.68	0.85	0.75
Quartile 3	0.84	0.77	0.88	0.78
Maximum	0.95	0.90	0.95	0.85

Abbreviations: MGB, Mass General Brigham; PSH, Penn State Health; VTE, venous thromboembolism.

^a^ Lower score indicates better performance.

Validation of DOVE eCQM MGB reviewed 146 DOVE eCQM cases, achieving a PPV of 97.26% (95% CI, 91.00%-99.00%), an NPV of 100.00% (95% CI, 95.00%-100.00%), and accuracy of 98.63% (95% CI, 95.00%-100.00%). PSH reviewed 47 cases, with PPV of 100.00% (95% CI, 85.00%-100.00%), NPV of 100.00% (95% CI, 87.00%-100.00%), and accuracy of 100.00% (95% CI, 92.00%-100.00%) (Table 3).

**Table 3.** Missed Opportunities for VTE Diagnosis Identified by Validation EHR Review

Variable	Cases, No. (%)	
	MGB (n = 73)	PSH (n = 25)
eCQM-negative cases, nondelayed VTE diagnosis		
Nondelayed cases in EHR review	73 (100.00)	25 (100.00)
Delayed cases in EHR review	0	0
eCQM-positive cases, delayed VTE diagnosis^a^		
Nondelayed cases in EHR review	2 (2.74)	0
Delayed cases in EHR review	71 (97.26)	22 (100.00)
Practitioner induced	59 (72.84)	11 (50.00)
Medical system induced	7 (8.64)	1 (4.55)
Patient	6 (7.41)	1 (4.55)
Unclassified delay	9 (11.11)	9 (40.91)

Abbreviations: eCQM, electronic clinical quality measure; EHR, electronic health record; MGB, Mass General Brigham; PSH, Penn State Health; VTE, venous thromboembolism.

^a^ Only 22 cases at PSH were included.

### EHR Review Findings

EHR reviews from both sites indicated that practitioner-related delays were the most frequent, followed by unclassified delays (Table 3). Practitioner-related delays often occurred when VTE symptoms present during a primary care visit were misattributed to a patient’s existing primary diagnosis or a chronic condition. Medical system delays included late-day computed tomography scan orders, causing follow-up scheduling delays. Patient-related delays occurred when recommendations to visit the emergency department or initiate treatment were not followed.

To further understand the nature of these delays, we hypothesized 3 types of VTE diagnosis: (1) VTE identified during the index visit but diagnosed after 24 hours, typically during hospital admission, (2) VTE not detected during the index visit but diagnosed during a subsequent office visit, and (3) unclassified delays with unclear origins. We then randomly selected and conducted 30 additional EHR reviews. In some cases, it was unclear whether the VTE developed gradually from symptoms noted during the index primary care visit (suggesting a possible physician-induced delay) or whether a new VTE had developed in the intervening period. Additional EHR reviews identified cases where patients exhibited VTE symptoms at the index visit that were not diagnosed, and subsequently experienced scenarios within 30 days that increased their risk for VTE. In these cases, it was challenging to determine whether the situation represented delayed VTE diagnosis or development of a new VTE due to new medical events, such as surgery, a fall, or taking a long-distance flight, all of which are known risk factors for VTE.^19,20,21^

### Thirty-Day All-Cause Mortality Calculation Based on the Time Frame to VTE Diagnosis

At MGB, 30-day all-cause mortality significantly increased as time to VTE diagnosis increased. Mortality increased from 17 deaths (2.52%) for diagnoses within 24 hours to 217 deaths (8.33%) for diagnoses after 24 hours, with an RR of 3.31 (95% CI, 2.03-5.38; *P* < .001). Similarly, at PSH, all-cause mortality increased from 2 deaths (4.65%) to 12 deaths (5.97%) with an RR of 1.28 (95% CI, 0.30-5.53; *P* = .73) (Table 4).

**Table 4.** Thirty-Day All-Cause Mortality by Time to VTE Diagnosis

Institution and time to VTE diagnosis	Cases, No. (%)^a^		Time to diagnosis, mean (SD), d	RR (95% CI)^b^	*P* value
	Total cases	Deaths within 30 d			
Mass General Brigham					
≤24 h	675 (100.00)	17 (2.52)	0.25 (0.12)	1.00 [Reference]	<.001
>24 h	2606 (100.00)	217 (8.33)	17.08 (8.75)	3.31 (2.03-5.38)	
Penn State Health					
≤24 h	43 (100.00)	2 (4.65)	0.33 (0.06)	1.00 [Reference]	.73
>24 h	201 (100.00)	12 (5.97)	12.44 (8.23)	1.28 (0.30-5.53)	

Abbreviations: RR, risk ratio; VTE, venous thromboembolism.

^a^ Percentages are calculated according to the 2 diagnostic time frames: timely diagnosis (≤24 hours) and delayed diagnosis of VTE.

^b^ RRs are calculated by comparing timely diagnosis to delayed diagnosis, with timely diagnosis serving as the reference group.

Across both time frames to VTE diagnosis (within and beyond 24 hours), the proportion of patients who received a diagnosis of PE was higher in the deceased patient group, whereas DVT or other types of embolism were more common among survivors (eTable 1 in Supplement 1). This analysis was reported for the MGB site only because of the small sample size of deceased patients at PSH. In addition, an expert EHR review of 54 deceased patient cases from both sites including both timely and delayed diagnoses was conducted. We found that all 12 cases diagnosed within 24 hours (timely diagnosis) had PE. Among these, 7 deaths were directly attributed to VTE (eTable 2 in Supplement 1). These findings suggest that diagnosis even within the 24-hour time frame is closely associated with PE diagnosis leading to death.

## Discussion

In this diagnostic study, we evaluated the feasibility of implementing a DOVE eCQM in 2 integrated care delivery systems. We also quantified the frequency of DOVE among patients who reported VTE symptoms during primary care visits. We found that the DOVE eCQM could be applied using different EHR vendor systems, as the necessary data elements were routinely available. We also found that the delayed VTE diagnosis rates were high across systems regardless of whether the greater than 24-hour or greater than 72-hour definitions were used, with all-cause mortality risk increasing with longer diagnostic delays. Practitioner-related factors were the most common cause of diagnostic delay.

The DOVE eCQM allows for systematic capture of VTE diagnostic delay, which has been a persistent challenge despite advancements in diagnostic methods. Our manual EHR review confirmed that nonspecific clinical signs and symptoms often hinder timely VTE recognition.^7,8^ This finding aligns with previous research^22^ suggesting that the absence of cardinal clinical features, such as chest pain or breathlessness, can contribute to delays in diagnosing PE. In EHR review, this was particularly evident when symptoms such as a cough overlapped with a patient’s underlying diagnosis (eg, lung cancer). To address these challenges, the DOVE eCQM uses a data-driven approach combined with clinical insights. We used NLP techniques to capture unstructured data, which are typically overlooked in clinical quality measures that primarily rely on structured data.

The absence of a standardized time frame for measuring VTE diagnostic delays complicates comparisons of delay rates across institutions and at the national level.^23^ As recommended by CMS, this study further explored VTE diagnostic delays by comparing rates using the greater than 24-hour and greater than 72-hour thresholds. DOVE rates remained consistently high under both definitions, and the risk of 30-day all-cause mortality increased from 2.52% to 8.33% as diagnostic delay lengthened. Our findings are comparable to the international 30-day mortality rates reported elsewhere^24^ for PE (4.98%) and lower limb DVT (2.52%). Study results showed that DVT diagnoses were more common among survivors, whereas PE diagnoses were more prevalent among patients who died. In addition, expert review confirmed that all 12 deceased patients who received a diagnosis within 24 hours had PE. This highlights early PE diagnosis as a critical factor in preventing death and reducing mortality rates. This is consistent with previous studies^25,26^ showing that PE has a significantly higher acute-phase mortality rate than DVT and that delayed PE diagnosis increases in-hospital mortality from 1.6% to 43.2% (within 24 hours vs between 24 hours and 30 days after emergency department presentation).^6^

Considering the association between delayed diagnosis and increased mortality and that the most common reason for delay is practitioner recognition of VTE (not securing a scan), we consulted with the Brigham and Women’s Hospital DOVE technical expert panel and recommend the greater than 24-hour definition of VTE diagnostic delay. Given the potential consequences of diagnostic delays, adopting this conservative time frame even with the small risk of false-positives seems justified to enhance patient safety in primary care settings. Our team presented these results in the spring of 2024 to the CMS committee, which included the DOVE eCQM on the 2024 Measures Under Consideration list. The DOVE eCQM was approved through the federal Medicare rule-making program January 2025.^27^

The DOVE eCQM leverages readily available EHR data, allowing health care practitioners to efficiently track and monitor VTE diagnostic delay. Its streamlined design enables integration into existing clinical workflows, providing a practical foundation for developing and implementing quality improvement initiatives aimed at reducing VTE diagnostic delays. Furthermore, benchmarking DOVE eCQM rates and integrating clinical decision support (CDS) systems in primary care may reduce VTE diagnostic delays, particularly those attributed to practitioner factors, which our study identified as a leading cause of delayed VTE diagnoses.

Although the DOVE eCQM is designed for measurement and benchmarking, CDS is also needed for identifying patients with vague or overlooked symptoms during primary care visits. By prompting earlier clinical suspicion of VTE, CDS can lead to timely recognition and follow-up actions, such as ordering a D-dimer test, and can reduce unnecessary reliance on costly imaging.^28,29,30^ Despite existing tools like the Wells score,^31^ diagnostic decisions often depend on practitioner judgment, making CDS a valuable aid. Together, CDS integration and ongoing measurement with the DOVE eCQM are important for reducing delays in VTE diagnosis. Measurement raises awareness of missed opportunities, whereas CDS facilitates earlier evaluation and more-efficient care in primary settings. We recommend expanding access to D-dimer testing, especially in rural clinics with limited access to definitive imaging. For low-risk patients, a negative result can safely rule out VTE and help avoid unnecessary, costly testing.^32,33^ Positive test results will stimulate prompt evaluation.

### Limitations

Our study has limitations. The delivery systems evaluated may differ from other integrated systems, although the 2 were in different regions, used different EHRs, and included practices in urban, metropolitan, and rural areas.

This study includes patients whose VTE symptoms were recorded in primary care, suggesting that asymptomatic patients were not captured. This study analyzed primary care visits only, so we lack data on the validity of applying this eCQM to specialty or other ambulatory practices.

The 24-hour diagnostic threshold was selected on the basis of expert guidance and CMS-aligned quality measurement standards. EHR review findings and mortality patterns support its clinical relevance, although future work will explore continuous time-to-diagnosis approaches.

Confounding and competing risks remain possible, and some deaths may not be attributable to the delayed diagnosis of VTE. Future research should consider more granular outcomes, such as VTE-specific mortality, or competing risk modeling to further isolate the associations of diagnostic delay with VTE-related outcomes. Furthermore, mortality data were obtained solely from EHR documentation and were not supplemented with external sources such as vital records or claims data, which may have led to underascertainment of deaths, particularly those occurring outside the health systems. Similarly, VTE events diagnosed at unaffiliated institutions may have been missed. These limitations could result in misclassification. However, both diagnostic groups were drawn from the same EHR populations, reducing the likelihood of differential bias in mortality comparisons.

Although the NLP platform is readily available, some sites may feel ill-prepared to adopt NLP for this measure.^13,34^ Nevertheless, the DOVE eCQM has the potential to enable continuous quality monitoring of delayed diagnosis of patients reporting VTE symptoms in primary care at institutional, regional, and national levels. Our future work involves expanding the DOVE eCQM to encompass other outpatient settings including urgent care and emergency departments and developing CDS to support timelier VTE diagnosis.

## Conclusions

In this diagnostic study of delayed diagnosis of VTE, we evaluated the DOVE eCQM, a systematically designed tool to measure delayed VTE diagnosis in primary care using EHR data. Such tools are needed to address diagnostic delays in outpatient settings. We validated the DOVE eCQM across 2 different EHR systems and found that delayed VTE diagnosis was associated with risk of higher all-cause mortality, underscoring the need for timely detection and treatment. The DOVE eCQM could provide a strong foundation for ongoing quality monitoring and improvement, ultimately advancing patient safety and outcomes.

## Notes

Supplement 1. **eTable 1.** Type of VTE categorized by time to VTE diagnosis based on *ICD* codes at MGB site **eTable 2.** Time to VTE and its association with 30-day VTE related mortality based on chart review **eAppendix 1.** Detailed description of the DOVE numerator and denominator **eAppendix 2.** Generalizability and feasibility of the NLP-based VTE symptom extraction tool

Supplement 2.
Data Sharing Statement

## References

[1] Beckman MG, Hooper WC, Critchley SE, Ortel TL. Venous thromboembolism: a public health concern. Am J Prev Med. 2010;38(4)(suppl):S495-S501. doi:10.1016/j.amepre.2009.12.017 20331949

[2] Perrier A, Roy PM, Aujesky D, . Diagnosing pulmonary embolism in outpatients with clinical assessment, D-dimer measurement, venous ultrasound, and helical computed tomography: a multicenter management study. Am J Med. 2004;116(5):291-299. doi:10.1016/j.amjmed.2003.09.041 14984813

[3] Tagalakis V, Patenaude V, Kahn SR, . Incidence of and mortality from venous thromboembolism in a real-world population: the Q-VTE Study Cohort. Am J Med. 2013;126(9):832.e13-832.e21. doi:10.1016/j.amjmed.2013.02.024 23830539

[4] Nijkeuter M, Söhne M, Tick LW, ; Christopher Study Investigators. The natural course of hemodynamically stable pulmonary embolism: clinical outcome and risk factors in a large prospective cohort study. Chest. 2007;131(2):517-523. doi:10.1378/chest.05-2799 17296656

[5] Hendriksen JM, Koster-van Ree M, Morgenstern MJ, . Clinical characteristics associated with diagnostic delay of pulmonary embolism in primary care: a retrospective observational study. BMJ Open. 2017;7(3):e012789. doi:10.1136/bmjopen-2016-012789 28279993 PMC5353317

[6] Mansella G, Keil C, Nickel CH, . Delayed diagnosis in pulmonary embolism: frequency, patient characteristics, and outcome. Respiration. 2020;99(7):589-597. doi:10.1159/000508396 32694258

[7] Pineda LA, Hathwar VS, Grant BJ. Clinical suspicion of fatal pulmonary embolism. Chest. 2001;120(3):791-795. doi:10.1378/chest.120.3.791 11555511

[8] Barais M, Morio N, Cuzon Breton A, . “I can’t find anything wrong: it must be a pulmonary embolism”: diagnosing suspected pulmonary embolism in primary care, a qualitative study. PLoS One. 2014;9(5):e98112. doi:10.1371/journal.pone.0098112 24840333 PMC4026480

[9] Dykes PC, Bowen M, Chang F, . Testing of an electronic clinical quality measure for diagnostic delay of venous thromboembolism (DOVE) in primary care. AMIA Annu Symp Proc. 2024;2023:339-348. 38222335 PMC10785865

[10] Partnership for Quality Measurement. Diagnostic delay of venous thromboembolism (DOVE) in primary care. 2023. Accessed August 19, 2025. https://p4qm.org/measures/3749e

[11] Centers for Medicare & Medicaid Services. Overview of the list of measures under consideration for December 1, 2023. Accessed August 19, 2025. https://mmshub.cms.gov/sites/default/files/2023-MUC-List-Overview.pdf

[12] Syrowatka A, Pullman A, Pajares E, . Accurately identifying incident cases of venous thromboembolism in the electronic health record: performance of a novel phenotyping algorithm. Thromb Res. 2024;243:109143. doi:10.1016/j.thromres.2024.109143 39303403

[13] Novoa-Laurentiev J, Bowen M, Pullman A, . An extraction tool for venous thromboembolism symptoms identification in primary care notes to facilitate electronic clinical quality measure reporting: algorithm development and validation. JMIR Med Inform. Preprint posted online June 28, 2024. doi:10.2196/63720 PMC12387394 40857675

[14] Partnership for Quality Measurement. Patient safety standing committee—spring 2023 measure evaluation meeting summary (version 1.0). August 21, 2023. Accessed August 19, 2025. https://p4qm.org/sites/default/files/2023-10/Spring-2023-Patient-Safety-Meas-Eval-Meeting-Summary-508.pdf

[15] Partnership for Quality Measurement. Full Measure Submission to PQM (Version 1.0). 2023. Accessed August 19, 2025. https://p4qm.org/EM/measure-submission

[16] Murphy DR, Zimolzak AJ, Upadhyay DK, . Developing electronic clinical quality measures to assess the cancer diagnostic process. J Am Med Inform Assoc. 2023;30(9):1526-1531. doi:10.1093/jamia/ocad089 37257883 PMC10436145

[17] Brunton NE, Wysokinski WE, Hodge DO, Vlazny DT, Houghton DE, Casanegra AI. Delayed anticoagulation in venous thromboembolism: reasons and associated outcomes. Res Pract Thromb Haemost. 2021;5(4):e12500. doi:10.1002/rth2.12500 34027287 PMC8117818

[18] Liu L, Bustamante R, Earles A, Demb J, Messer K, Gupta S. A strategy for validation of variables derived from large-scale electronic health record data. J Biomed Inform. 2021;121:103879. doi:10.1016/j.jbi.2021.103879 34329789 PMC9615095

[19] Khan F, Tritschler T, Kahn SR, . Venous thromboembolism. Lancet. 2021;398(10294):64-77. doi:10.1016/S0140-6736(20)32658-1 33984268

[20] Neeman E, Liu V, Mishra P, . Trends and risk factors for venous thromboembolism among hospitalized medical patients. JAMA Netw Open. 2022;5(11):e2240373. doi:10.1001/jamanetworkopen.2022.40373 36409498 PMC9679881

[21] Pastori D, Cormaci VM, Marucci S, . A comprehensive review of risk factors for venous thromboembolism: from epidemiology to pathophysiology. Int J Mol Sci. 2023;24(4):3169. doi:10.3390/ijms24043169 36834580 PMC9964264

[22] Goyard C, Côté B, Looten V, . Determinants and prognostic implication of diagnostic delay in patients with a first episode of pulmonary embolism. Thromb Res. 2018;171:190-198. doi:10.1016/j.thromres.2018.08.015 30190113

[23] van Maanen R, Trinks-Roerdink EM, Rutten FH, Geersing GJ. A systematic review and meta-analysis of diagnostic delay in pulmonary embolism. Eur J Gen Pract. 2022;28(1):165-172. doi:10.1080/13814788.2022.2086232 35730378 PMC9246192

[24] RIETE Registry. Death within 30 days. 2024. Accessed December 18, 2024. https://rieteregistry.com/graphics-interactives/dead-30-days/

[25] Spencer FA, Gore JM, Lessard D, Douketis JD, Emery C, Goldberg RJ. Patient outcomes after deep vein thrombosis and pulmonary embolism: the Worcester Venous Thromboembolism Study. Arch Intern Med. 2008;168(4):425-430. doi:10.1001/archinternmed.2007.69 18299499 PMC2762782

[26] Naess IA, Christiansen SC, Romundstad P, Cannegieter SC, Rosendaal FR, Hammerstrøm J. Incidence and mortality of venous thrombosis: a population-based study. J Thromb Haemost. 2007;5(4):692-699. doi:10.1111/j.1538-7836.2007.02450.x 17367492

[27] Centers for Medicare & Medicaid Services. 2024 CMS Measures Under Consideration (MUC) list supporting materials. 2025. Accessed April 1, 2025. https://mmshub.cms.gov/measure-lifecycle/measure-implementation/pre-rulemaking/lists-and-reports/2024-MUC-List-materials

[28] Righini M, Perrier A, De Moerloose P, Bounameaux H. D-dimer for venous thromboembolism diagnosis: 20 years later. J Thromb Haemost. 2008;6(7):1059-1071. doi:10.1111/j.1538-7836.2008.02981.x 18419743

[29] Oudega R, Moons KG, Hoes AW. Ruling out deep venous thrombosis in primary care: a simple diagnostic algorithm including D-dimer testing. Thromb Haemost. 2005;94(1):200-205. doi:10.1160/TH04-12-0829 16113804

[30] Kruip MJ, Slob MJ, Schijen JH, van der Heul C, Büller HR. Use of a clinical decision rule in combination with D-dimer concentration in diagnostic workup of patients with suspected pulmonary embolism: a prospective management study. Arch Intern Med. 2002;162(14):1631-1635. doi:10.1001/archinte.162.14.1631 12123408

[31] Wells PS, Anderson DR, Rodger M, . Derivation of a simple clinical model to categorize patients probability of pulmonary embolism: increasing the models utility with the SimpliRED D-dimer. Thromb Haemost. 2000;83(3):416-420. doi:10.1055/s-0037-1613830 10744147

[32] Wells PS, Anderson DR, Rodger M, . Excluding pulmonary embolism at the bedside without diagnostic imaging: management of patients with suspected pulmonary embolism presenting to the emergency department by using a simple clinical model and d-dimer. Ann Intern Med. 2001;135(2):98-107. doi:10.7326/0003-4819-135-2-200107170-00010 11453709

[33] Price CP, Fay M, Hopstaken RM. Point-of-care testing for d-dimer in the diagnosis of venous thromboembolism in primary care: a narrative review. Cardiol Ther. 2021;10(1):27-40. doi:10.1007/s40119-020-00206-2 33263839 PMC8126530

[34] Dykes PC, Bates D, Chang F, . Using structured and unstructured EHR features to quantify delayed diagnosis of venous thromboembolism (VTE) in primary care: a multi-site study. Paper presented at: AMIA Informatics Submit 2024;453. Accessed August 25, 2025. https://s4.goeshow.com/amia/summit/2024/schedule_at_a_glance.cfm

