# Supplementary material for: Delayed Venous Thromboembolism Diagnosis and Mortality Risk
Source: JAMA Netw Open. 2025 Sep 26;8(9):e2533928. doi: 10.1001/jamanetworkopen.2025.33928 (PMC12475950; doi:10.1001/jamanetworkopen.2025.33928)
Supplement: Supplement 1. — eTable 1. Type of VTE categorized by time to VTE diagnosis based on ICD codes at MGB site eTable 2. Time to VTE and its association with 30-day VTE related mortality based on chart review eAppendix 1. Detailed description of the DOVE numerator and denominator eAppendix 2. Generalizability and feasibility of the NLP-based VTE symptom extraction tool [file jamanetwopen-e2533928-s001.pdf]

Supplemental Online Content

Kang MJ, Schreiber R, Baris VK, et al. Delayed venous thromboembolism diagnosis and mortality risk. *JAMA Netw Open*. 2025;8(9):e2533928.
doi:10.1001/jamanetworkopen.2025.33928

eTable 1. Type of VTE categorized by time to VTE diagnosis based on *ICD* codes at MGB site

eTable 2. Time to VTE and its association with 30-day VTE related mortality based on chart review

eAppendix 1. Detailed description of the DOVE numerator and denominator

eAppendix 2. Generalizability and feasibility of the NLP-based VTE symptom extraction tool

This supplemental material has been provided by the authors to give readers additional information about their work.

© 2025 Kang MJ et al. *JAMA Network Open*.

eTable 1. Type of VTE categorized by time to VTE diagnosis based on *ICD* codes at MGB site.

Type of VTE	Time to VTE Diagnosis					
	≤24 hours			>24 hours		
	Death n(%)	No Death n(%)	Total n(%)	Death n(%)	No Death n(%)	Total n(%)
Total	17 (2.52)	658 (97.48)	675 (100.00)	217 (8.33)	2389 (91.67)	2606 (100.00)
PE	11 (64.71)	230 (34.95)		123 (56.68)	1130 (47.30)	
Non-PE	6 (35.29)	428 (65.05)		94 (43.32)	1259 (52.70)	

ICD: International Classification of Disease; PE: pulmonary embolism; Non-PE: deep vein thrombosis or other types of embolism.

© 2025 Kang MJ et al. *JAMA Network Open*.

eTable 2. Time to VTE and its association with 30-day VTE related mortality based on chart review

Time to VTE Diagnosis	Study site	Total	VTE related mortality		Type of VTE (PE, DVT, or both)
		Chart review cases (n)	VTE related death, n(%)	VTE related death, 95% CI	
≤24 hours	MGB	10	6 (60.00)	31-84%	PE: 8<br>Both:2
	PSH	2	1 (50.00)	3-97%	PE: 2
>24hours	MGB	30	15 (50.00)	33-67%	PE: 18<br>DVT:11<br>Both:1
	PSH	12[a]	8 (72.73)	43-91%	PE: 3<br>DVT:5<br>Both:3

[a] One case could not be categorized by VTE type or its relation to the cause of death as it did not meet study criteria. This case had no VTE-related symptoms during the primary care visit and was considered a false positive for the DOVE eCQM.

The classification of VTE diagnostic delay was based on information recorded in clinical notes, which differ from ICD-based diagnoses, and overlapping diagnoses were permitted. ICD: International Classification of Disease; PE: pulmonary embolism; DVT: deep vein thrombosis.

© 2025 Kang MJ et al. *JAMA Network Open*.

eAppendix 1: Detailed description of the DOVE numerator and denominator[1]

Figure 1: Process for Identifying Eligible Patients and Calculating the DOVE eCQM Rate

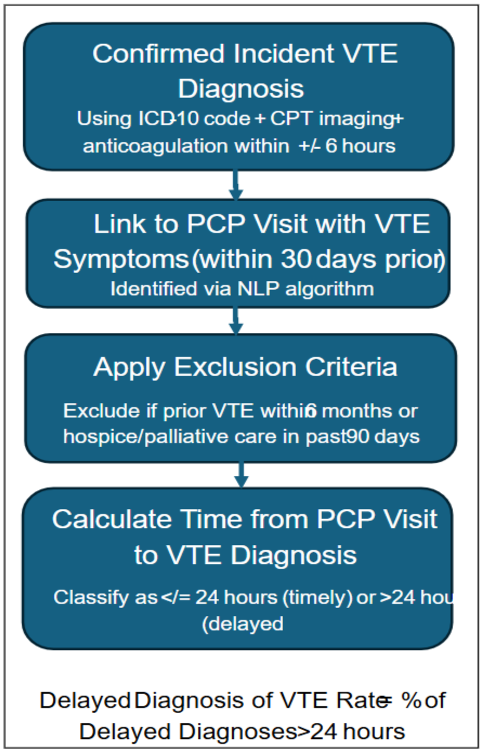

To identify eligible patients for the DOVE eCQM, we applied a multi-step process combining a validated phenotyping algorithm and EHR-based symptom and visit linkage (**Figure 1**):

1. **Identification of Incident VTE Diagnoses:** We used a high-performance phenotyping algorithm that required *three concurrent EHR indicators* to confirm incident VTE:
    a) An ICD-10 diagnostic code for VTE
    b) A CPT code for VTE-related imaging, and
    c) New anticoagulant prescription or order within ±6 hours of the VTE related imaging. Encounters were excluded if any VTE diagnosis was identified in the prior 6 months, ensuring only new (incident) cases were included.
2. **Linkage to Prior Primary Care Visit with Symptoms:** For each confirmed VTE case, we looked back 30 days to identify a primary care provider visit with documented VTE-related signs and or symptoms in the visit notes using a natural language processing algorithm[i] capable of detecting predefined signs/symptoms (See VTE Symptom Lexicon below). The earliest such visit was labeled the *index visit*.

Table 1: VTE Symptom Lexicon.

Location-Independent Symptoms	Location-Dependent Symptoms	Locations
cough	pain	chest*
hypotension	numbness	calf
lightheadedness	tingling	leg
shortness of breath	redness	foot
syncope	swelling	
tachycardia	tenderness	
hemoptysis	warmth	

*The “chest” location was only used when searching for the “pain” symptom.

© 2025 Kang MJ et al. *JAMA Network Open*.

3. **Application of Inclusion and Exclusion Criteria:**
   Patients were included in the denominator if they were ≥18 years old, had an incident VTE diagnosis within 30 days of the index visit, and had no VTE in the prior 6 months. We excluded patients receiving palliative or hospice care in the 90 days before the index visit to avoid misclassification related to end-of-life care priorities.

4. **Delayed Diagnosis of VTE (DOVE) Rate Definition:**
   The DOVE rate represents the proportion of patients in the denominator whose VTE diagnosis occurred more than 24 hours after the index visit, indicating delayed recognition.

[1] Syrowatka A, Pullman A, Pajares E, et al. Accurately identifying incident cases of venous thromboembolism in the electronic health record: Performance of a novel phenotyping algorithm. Thrombosis Research. 2024;243:109143.

© 2025 Kang MJ et al. *JAMA Network Open*.

eAppendix 2. Generalizability and feasibility of the NLP-based VTE symptom extraction tool[1]

This eSupplement provides additional detail on the technical implementation, replicability, and generalizability of VTExt, the natural language processing (NLP) tool used in the DOVE eCQM to extract VTE symptoms from unstructured clinical notes.

1. NLP Platform and Implementation

- The tool was implemented using Python and is built upon the Medical Text Extraction, Reasoning and Mapping System (MTERMS).
- VTExt uses a rule-based extraction approach, guided by a clinician-reviewed lexicon of VTE symptoms.
- Regular expressions were used to match symptom and location terms, while contextual rules accounted for negations and clinical modifiers.
- A streamlined version was developed for clinical use that returns a binary output indicating whether any VTE symptom was present in a note.

2. Open Access and Transparency

5. VTExt is not proprietary. Pseudocode and setup instructions are available publicly at: https://github.com/jnlaurentiev/dove_vtext
6. Its rule-based logic ensures full transparency and ease of audit compared to black-box machine learning models.

3. External Validations and Portability

- VTExt was validated using clinical notes from two external healthcare systems:
  - University of Kentucky (Veradigm EHR)
  - Penn State Health (Oracle Cerner EHR)
- Both sites used different EHR vendors and served diverse populations (urban, rural, mixed).
- Despite this heterogeneity, VTExt achieved high precision (PPV = 1.00 at UK; 0.98 at PSH) and high specificity (1.00 at UK; 0.99 at PSH).
- The tool functioned consistently across EHR platforms once text notes were extracted, demonstrating strong portability and generalizability.

© 2025 Kang MJ et al. *JAMA Network Open*.

4. Constraints on Replicability

- Key requirements for replication include:
  - Access to free-text clinical notes
  - Ability to query and preprocess text data from local EHR systems
  - Basic NLP proficiency to configure the tool and adapt the lexicon if necessary
- Once note access is established, no proprietary software or specialized infrastructure is needed to run VTExt.

5. Summary

VTExt represents a practical, open-source solution for extracting clinically meaningful information from unstructured notes to support eCQM implementation. It has demonstrated robust performance in internal and external settings and is feasible to replicate and adapt in other health systems.

[1] Novoa-Laurentiev J, Bowen M, Pullman A, Song, W, Syrowatka, A, Chen J, Sainlaire M, Chang, F, Gray K, Purushottam P, Liu, L, Nawab K, Hijjawi S, Schreiber R, Zhou, L, Dykes, PC. An Extraction Tool for Venous Thromboembolism Symptom Identification in Primary Care Notes to Facilitate Electronic Clinical Quality Measure Reporting: Algorithm Development and Validation. JMIR Med Inform (in press). doi:10.2196/63720, http://dx.doi.org/10.2196/63720.

© 2025 Kang MJ et al. *JAMA Network Open*.
