# Supplementary material for: Delayed Venous Thromboembolism Diagnosis and Mortality Risk
Source: JAMA Netw Open. 2025 Sep 26;8(9):e2533928. doi: 10.1001/jamanetworkopen.2025.33928 (PMC12475950; doi:10.1001/jamanetworkopen.2025.33928)
Supplement: Supplement 2. — Data Sharing Statement [file jamanetwopen-e2533928-s002.pdf]

Data Sharing Statement

Kang. Delayed Venous Thromboembolism Diagnosis and Mortality Risk. *JAMA Netw Open*. Published September 26, 2025. doi:10.1001/jamanetworkopen.2025.33928

Data
Data available: No
